# A Large Ascending Aorta Thrombus in a Patient with Acute Myocardial Infarction—Case Report

**DOI:** 10.3390/medicina57111176

**Published:** 2021-10-28

**Authors:** Horațiu Moldovan, Cristian Bulescu, Andra-Mădălina Sibișan, Robert Țigănașu, Cătălin Cacoveanu, Claudia Nica, Andreea Rachieru, Daniela Gheorghiță, Ondin Zaharia, Șerban Bălănescu, Alexandru Scafa-Udriște

**Affiliations:** 1 Faculty of Medicine and Pharmacy, Carol Davila University of Medicine and Pharmacy, 050474 Bucharest, Romania; h_moldovan@hotmail.com (H.M.); cristianbulescu@gmail.com (C.B.); ondin.zaharia@gmail.com (O.Z.); smbala99@hotmail.com (Ș.B.); alexscafa@yahoo.com (A.S.-U.); 2 Department of Cardiovascular Surgery, Clinical Emergency Hospital Bucharest, 014461 Bucharest, Romania; andrasibisan@yahoo.com (A.-M.S.); tiganasu.robert@yahoo.com (R.Ț.); catalin.cacoveanu@gmail.com (C.C.); bianca.nica@yahoo.com (C.N.); 3 Ponderas Academic Hospital, 014142 Bucharest, Romania; rachieru_andreea@yahoo.com; 4 Faculty of Materials Science and Engineering, Politehnica University of Bucharest, 060042 Bucharest, Romania; 5 Prof. Dr. Theodor Burghele Clinical Hospital, 050659 Bucharest, Romania

**Keywords:** large thrombus, ascending aorta, acute myocardial infarction, surgical thrombectomy

## Abstract

We present the case of a 50-year-old male, with no cardiovascular risk factors other than smoking, that presented with acute chest pain, revealed to be an acute myocardial infarction with a large thrombus located in the ascending aorta. Such findings are rare in a patient with no other afflictions, such as atherosclerosis, aortic aneurysm, or aortic wall injury (surgical or traumatic). There is no specific pathway regarding the management of ascending aorta thrombus in such a patient; therapeutic options include surgical, interventional, or medical methods. Surgical thrombectomy was performed in this case, considering the high risk of systemic embolism and stroke and the hemodynamic stability of the patient.

## 1. Introduction

Aortic thrombosis is rendered as the cause of 5% of systemic embolisms [1]; however, an aortic thrombus is rarely encountered and is seldomly diagnosed before embolic complications. The etiology of thoracic aortic thrombosis is usually connected to a previous hematological disorder resulting in a hypercoagulability state [2,3], with other causes including aortic pathologies, such as aneurysm, dissection, or vasculitis. The treatment options for aortic thrombosis are medical, interventional, or the surgical approach. The usually proposed course of treatment for aortic thrombosis is either surgical thrombectomy after anticoagulation therapy, prolonged oral anticoagulation, or both. When referring to the ascending aorta, although rare, the most preferred means of treatment is the surgical removal of the thrombus [4].

## 2. Case Report

**Figure 1:**
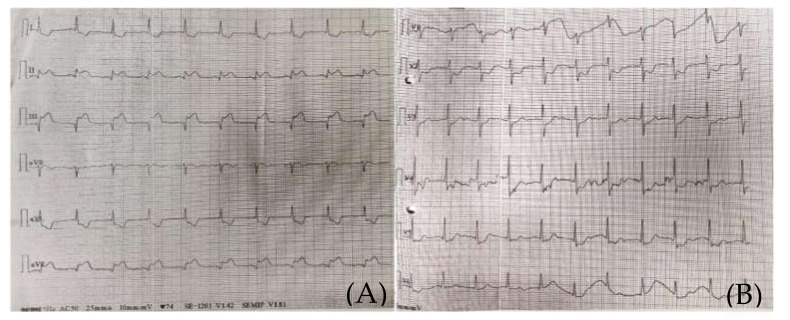
(**A**) (standard limb leads) and (**B**) (standard precordial leads). Emergency room 12-Lead ECG presenting an inferior myocardial infarction, ST-T segment elevation.

A 50-year-old male, with no known cardiovascular risk factors other than smoking, presented with acute chest pain, vision impairment, and restlessness and was admitted to the hospital. The ECG (electrocardiography) anomalies were consistent with an inferior acute ST elevation myocardial infarction (Figure 1). Accordingly, blood tests revealed troponin elevation (2.6 ng/mL—ULN < 0.021 ng/mL), 262,000 platelets/μL, 14,000 leukocytes/μL, CK-MB—on admission day—44 U/L, and 377 U/L the following day.

**Figure 2:**
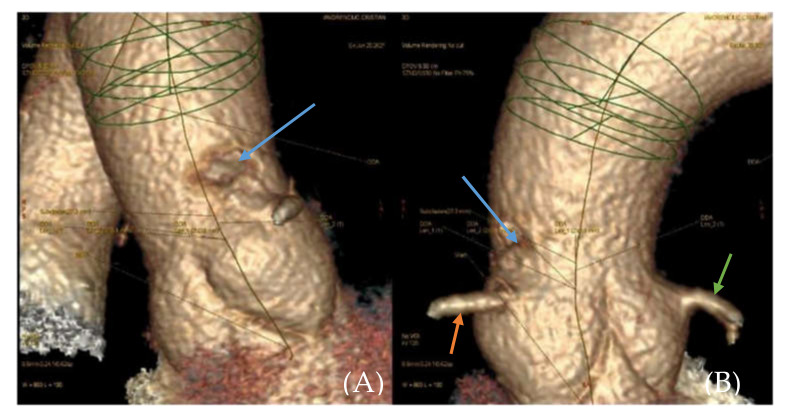
(**A**,**B**). 3D reconstruction of CTA(computed tomography angiography) image of the ascending aorta. Filling defect in the ascending aorta (blue arrow). Right coronary artery (orange arrow). Left coronary artery (green arrow).

**Figure 3:**
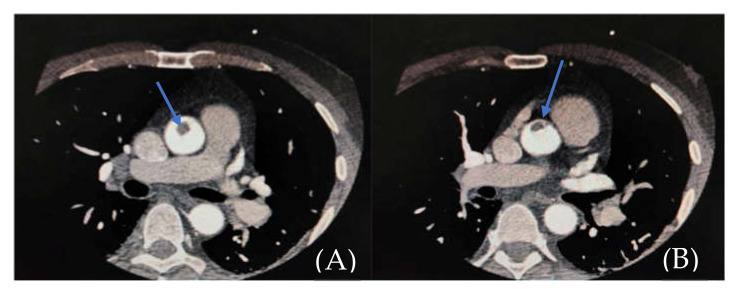
(**A**,**B**). CTA showing endoluminal aortic thrombus (filling defect in the ascending aorta—blue arrow).

The clinical and paraclinical diagnosis suggested an inferior myocardial infarction with ST-T segment elevation (Figure 1A,B); according to the national protocol [5], a loading dose of aspirin, ticagrelor, and heparin was administered. A first echocardiography exam showed a thickened anterior aortic wall and a small amount of pericardial fluid. In this context, and due to neurological symptoms with vision impairment, significant restlessness, and mild confusion, aortic dissection was suspected, so thoracic and cerebral CT (computer-tomography) angiography was performed. It found a floating thrombus in the ascending aorta, located in the proximity of the right coronary ostium, with a 4-mm base and 5-mm thickness (Figure 2 and Figure 3). There were no other atherosclerotic deposits in the aortic root or ascending aorta. There were no initial signs of cerebral lesions by CT scanning.

**Figure 4:**
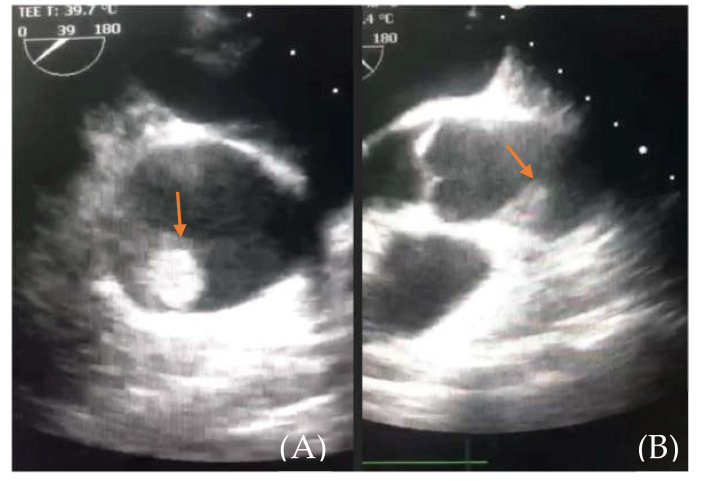
(**A**) (thrombus—orange arrow) and (**B**) (aortic root and thrombus). Emergency room transesophageal echocardiography showing the floating thrombus located in the ascending aorta.

The patient was then referred to our center for emergency treatment. Transesophageal echocardiography confirmed an approximately 2 cm, highly mobile mass floating in the ascending aorta, seemingly inserted in the right sino-tubular junction, with a high embolic risk (Figure 4). The right Valsalva sinus walls were layered with a mass with small, thin, highly mobile extensions. The infero-septal wall of the left ventricle was akinetic at the base, while the inferior and infero-lateral walls were hypokinetic (RCA-right coronary artery territory); LVEF (left ventricle ejection fraction) was assessed at 40–45%. No other cardiac masses were found nor any significant valvular disease.

**Figure 5:**
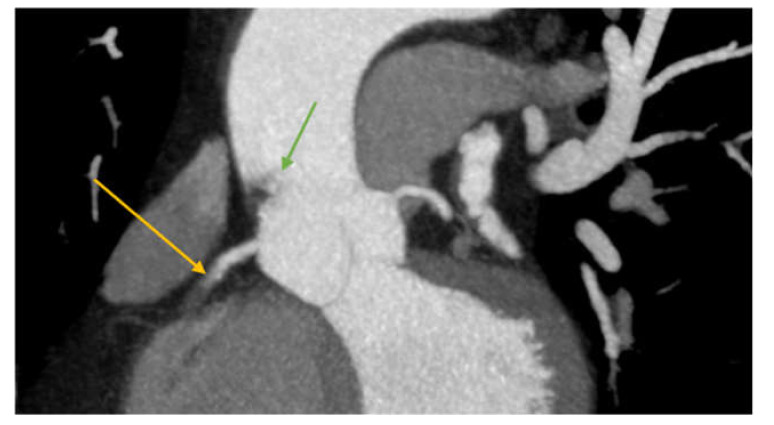
CTA image showing right coronary artery obstruction (yellow arrow) and the endoluminal thrombus (filling defect in the ascending aorta—green arrow).

Given the dimensions, location, and high mobility of the mass, with an extremely high embolic risk, the decision was made for surgical embolectomy. Additionally, due to the position of the thrombus in the aortic root, coronary angiography was not performed in order to avoid dislodging the thrombus. Instead, a coronary computed tomography angiography was performed, which showed an occlusion in the second segment of the RCA (Figure 5).

**Figure 6:**
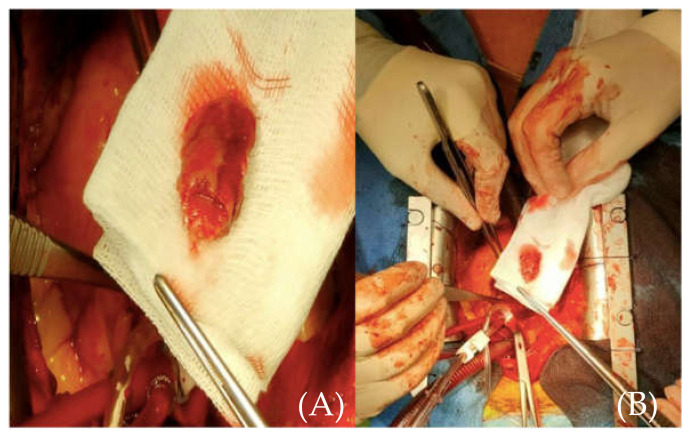
(**A**) (close-up image of the thrombus) and (**B**) (size referencing) intraoperative aspect of the removed thrombus.

Emergency surgery was performed ten hours from chest pain onset after full non-invasive assessment of the brain, thoracic aorta, the heart, and coronary arteries. The approach was through a median sternotomy, followed by central cannulation, with cardio-pulmonary bypass, aortic cross clamp, and aortic antegrade cardioplegia administration. A transverse aortotomy was performed at the level of the sino-tubular junction, and a floating 2/3-cm thrombus was discovered adjacent to the origin the RCA and easily removed en bloc (Figure 6). It showed macroscopic signs of different stages of evolution. There were no alterations of the wall of the ascending aorta or the aortic root and no atherosclerotic deposits.

The aorta was then closed in the usual fashion, and the anastomosis of a saphenous vein graft was performed on the RCA and then on the ascending aorta. The aorta was unclamped, and the patient was slowly weaned off cardiopulmonary bypass, with a 20-min circulatory assist time. The cardio-pulmonary bypass time was 85 min, and the aortic cross-clamping time was 48 min.

**Figure 7:**
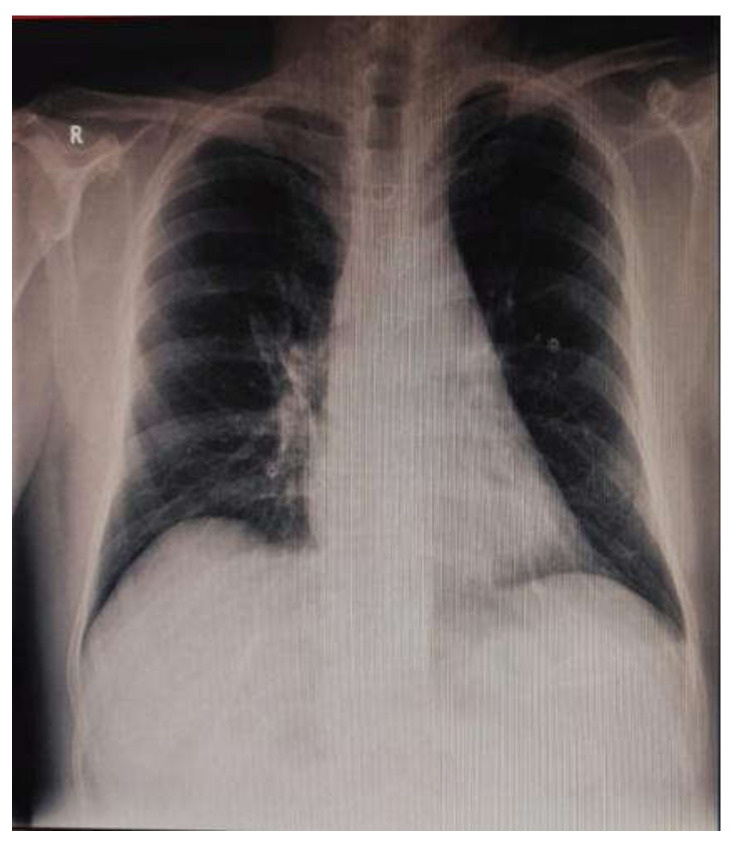
Postoperative chest X-ray.

In the postoperative period, the patient maintained a dysfunctional, nondilated right ventricle and a moderate LV dysfunction (LVEF 35%). High doses of Dobutamine and small doses of Noradrenalin were necessary in the first 8 postoperative days. Otherwise, the patient was extubated on POD1 and showed no neurological or other end-organ deficit. The chest tube drainage was minimal. The postoperative chest X-ray was normal (Figure 7). The LV function improved under standard, guideline-prescribed pharmacological treatment (beta-blockers, ACE-inhibitors, digoxin, statin, aspirin, and clopidogrel).

Discharge echocardiography showed an improvement of the right ventricular dysfunction, with a VTI of 12 cm/s and a RV/RA gradient of 36 mmHg, hypokinesis of the anterior and inferior portions of the interventricular septum and the posterior wall of the RV, and slight dyskinesis of the basal half of the RV inferior wall. The left ventricular function improved (LVEF 45%, subaortic VTI 18.2 cm), and there was a moderate mitral regurgitation with an ischemic mechanism.

**Figure 8:**
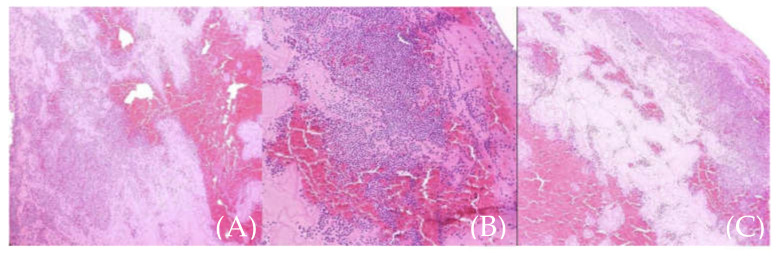
(**A**–**C**) (different sections of the same sample). Microscopic aspect of the thrombus (fibrin and white and red blood cells).

Microscopic examination of the extracted aortic mass showed amorphous thrombotic content; no evidence of malignant components were found; it was composed only of fibrin and white and red blood cells (Figure 8).

The patient was examined for coagulation anomalies and was diagnosed with antiphospholipid syndrome LA1/LA2—2.38/s—LA2—56.4/s and protein C and S deficit, with MTHFR gene mutation (A1298C-positive homozygous genotype), findings that explain the thrombotic risk that the patient had prior to this event. In spite of the newly established diagnosis, the patient had no previous thrombotic events.

The patient was discharged fourteen days after the surgery, with normal CK and CK-MB values. The treatment included double antiplatelet therapy for one month, with clopidogrel and aspirin associated with oral anticoagulation with acenocoumarol, later to be left only with clopidogrel and acenocoumarol for 1 year. The INR at discharge was 3.5. The follow-up echocardiography and cardiology exam were scheduled for the next month, and cardiology check-ups were recommended 3, 6, and 12 months after discharge. In addition, a second thrombophilic profile and an autoimmune disease screening were recommended and later confirmed the antiphospholipid syndrome associated with increased levels of homocysteine (17 mcmol/L). The first follow-up showed normal clinical examination, right ventricle disfunction with akinetic free right ventricle wall, and normal D-Dimer values.

## 3. Discussion

With the increase in availability of non-invasive diagnostic tools for the evaluation of the ascending aorta, such as transesophageal echocardiography (TEE) and computed tomography angiography (CTA), an increase in the diagnosis of mobile aortic thrombi has been seen in the last decade [6]. The guidelines for STEMI (ST-segment elevation myocardial infarction) determine the course of a patient with this ailment and send him directly to the angiography room for a PCI (percutaneous coronary intervention), but in this case, the echocardiography was the treatment-shifting investigation, proving its merits as a screening method that should be instated in the case of myocardial infarction, specifically in atypical STEMI. Furthermore, a CT exam can and should be performed if the suspicion for aortic pathology or embolic stroke is raised. The discovery of an aortic mass opens up the debate as to how to classify it: a thrombus, as the case was here; a tumor; or a vegetation (marantic or infectious). Although there are certain characteristics that are found through echocardiography and can make the distinction between these entities (location, morphology, clinical presentation, valve excursion), the final say is that of the histopathological analysis [7].

Differential diagnosis of acute inferior myocardial infarction can and should always be made with type A aortic dissection [8], especially when neurological manifestations are associated. Acute Stanford type A aortic dissection complicated with acute myocardial infarction is a rare but devastating event that occurs in approximately 3% of patients with aortic dissection [9]. In most patients, the right coronary artery is most frequently involved since dissection more commonly originates from the right anterior aspect of the ascending aorta, above the right sinus of Valsalva [10]. A correct initial diagnosis between these two diseases must be made because the first-line treatment is also different: the guideline for patients with ST-segment elevation myocardial infarction dictates a rapid primary percutaneous coronary intervention with a door-to-balloon time of less than 90 min and antiplatelet therapy given at the time of first medical contact [11]. In patients with type A aortic dissection misdiagnosed with acute myocardial infarction, a situation occurring in approximately 30% of cases [12], the inappropriate treatment with antiplatelet, antithrombin, and thrombolytic agents can aggravate the surgical prognosis by causing severe bleeding. Our patient presented with acute chest pain, visual impairment, and ECG abnormalities consistent with an acute inferior myocardial infarction. Aortic dissection was suspected, so thoracic and cerebral CT angiography was performed, which did not confirm the suspected aortic dissection, but it did find a floating thrombus in the ascending aorta.

A thrombus located in the proximal portion of the ascending aorta may be the cause for acute coronary syndrome, either by direct obstruction of the coronary ostium or distal coronary emboli [13]. In such an exceptional case, where aortic thrombosis is found without the association of aortic root pathology (aneurysm, dissection, vasculitis), a discussion should be made as to whether the thrombosis of the aorta is the cause of right coronary embolism (which seems to be the line of events in this case) or thrombosis of the right coronary extended in the aortic root.

Thrombi formation in a normal aorta is an uncommon and life-threatening occurrence due to the high risk of distal thromboembolic events [14]. The exact mechanism of thrombi formation is not fully understood. The scarcity of the occurrence stands in the high blood flow and sheer stress of the aorta [15]. Non-atherosclerotic aortic thrombi have been linked to either aortic pathology or coagulation disturbances. In this case, the patient was diagnosed with antiphospholipid syndrome, deficit of both C and S protein, with positive MTHFR mutation, which are hypercoagulative states that significantly increase the risk for thrombus development in both venous and arterial territory, though only a few cases are known to be associated with aortic thrombosis [2,3].

The antiphospholipid syndrome is characterised by thrombotic manifestations, most often arterial but rarely aortic. Reviewing the literature, we discovered a similar case described by Letang E et al., where the patient was similar in age and autoimmune profile, with no cardiovascular risk factors other than smoking and with no atherosclerotic deposits on the aortic wall but different in sex (female) and medical history (thrombotic events that lead to the indication of treatment with oral anticoagulation). Given her history with anticoagulation, the patient received only anticoagulation therapy with a good outcome [16]. Another case described by G. M. M. Shahin et al. is of a female patient similar in age and risk factors (smoking) and no atherosclerotic deposits but with a different autoimmune profile (heterozygote for factor V Leiden). The patient was managed surgically in this case [13]. Therefore, we can establish the importance of the autoimmune profile in discovering the aetiology of the ailment in patients with no medical history and aortic thrombosis and in deciding the appropriate management of such a case. The scarcity of aortic thrombosis in an aorta with no atherosclerotic deposits makes it impossible to define a guideline treatment with both prevention of future thrombosis using antiplatelet and/or anticoagulation therapy and other treatment option, leaving experience to dictate the course of treatment.

Discussing the best course of treatment for an ascending aortic thrombus, there are some aspects that should be taken into consideration. Usually, the initial proposed course of treatment is either surgical thrombectomy after anticoagulation therapy, prolonged oral anticoagulation, or both. The location of the thrombus is a major decisive factor when choosing the appropriate method of treatment. The presence of a thrombus in the ascending aorta has often led to the surgical approach [3], while endovascular [17] or medical treatments were preferred for thrombi located in the aortic arch and descending and abdominal aorta [18,19]. Furthermore, the hemodynamic stability is also a deciding factor. In our case, the patient’s hemodynamic stability allowed a safe surgical thrombectomy. An unstable patient requires a more conservative approach, including anticoagulation therapy or percutaneous interventions [20]. The timing or indication for surgical thrombectomy are still controversial, the operative risk being perceived as disproportionately high as opposed to the potential benefit. Furthermore, an unknown factor is the risk of recurrent embolic events after anticoagulation therapy, with few reports showing the dissolution of the thrombus with anticoagulation therapy alone [21,22].

## 4. Conclusions

A large thrombus located in the ascending aorta in a patient with acute myocardial infarction is a delicate situation that has no specific management plan. The experience of the surgeon, location of the thrombus, and the general status of the patient were the decisive factors that guided the treatment path. The most useful examinations in this case was the echocardiography and CTA, which revealed its existence and specific location of the thrombus and its dimensions. Surgical thrombectomy was performed, this being the method that significantly reduces the risk of embolism, be it systemic or cerebral, and what we believe to be the best course of treatment.

## Data Availability

The study did not report any data.
